# Chloroplast Phylogenomic Analyses Reveal a Maternal Hybridization Event Leading to the Formation of Cultivated Peanuts

**DOI:** 10.3389/fpls.2021.804568

**Published:** 2021-12-17

**Authors:** Xiangyu Tian, Luye Shi, Jia Guo, Liuyang Fu, Pei Du, Bingyan Huang, Yue Wu, Xinyou Zhang, Zhenlong Wang

**Affiliations:** ^1^School of Life Sciences, Zhengzhou University, Zhengzhou, China; ^2^Key Laboratory of Oil Crops in Huang-Huai-Hai Plains, Ministry of Agriculture and Rural Affairs, Henan Provincial Key Laboratory for Oil Crops Improvement, Henan Institute of Crop Molecular Breeding, Henan Academy of Agricultural Sciences, Zhengzhou, China

**Keywords:** *Arachis*, whole plastid genome, genetic structure, phylogenomics, maternal hybridization event

## Abstract

Peanuts (*Arachis hypogaea* L.) offer numerous healthy benefits, and the production of peanuts has a prominent role in global food security. As a result, it is in the interest of society to improve the productivity and quality of peanuts with transgenic means. However, the lack of a robust phylogeny of cultivated and wild peanut species has limited the utilization of genetic resources in peanut molecular breeding. In this study, a total of 33 complete peanut plastomes were sequenced, analyzed and used for phylogenetic analyses. Our results suggest that sect. *Arachis* can be subdivided into two lineages. All the cultivated species are contained in Lineage I with AABB and AA are the two predominant genome types present, while species in Lineage II possess diverse genome types, including BB, KK, GG, *etc*. Phylogenetic studies also indicate that all allotetraploid cultivated peanut species have been derived from a possible maternal hybridization event with one of the diploid *Arachis duranensis* accessions being a potential AA sub-genome ancestor. In addition, *Arachis monticola*, a tetraploid wild species, is placed in the same group with all the cultivated peanuts, and it may represent a transitional species, which has been through the recent hybridization event. This research could facilitate a better understanding of the taxonomic status of various *Arachis* species/accessions and the evolutionary relationship among them, and assists in the correct and efficient use of germplasm resources in breeding efforts to improve peanuts for the benefit of human beings.

## Introduction

The genus *Arachis* consists of approximately 81 species, which represent nine sections and 16 genome types, and are mainly distributed in the tropics and subtropics of South America (Stalker, 2017). Among these, peanut or groundnut (*Arachis hypogaea* L.) is a world-famous legume crop and cultivated by more than one hundred countries in the tropical and subtropical regions (Singh and Moss, 1982; Varshney et al., 2009; Pandey et al., 2020). Peanut was domesticated about 3,500–9400 years ago in South America (Bertioli et al., 2019; Chen et al., 2019; Zhuang et al., 2019). It is known as the “longevity fruit,” “poor man’s almonds” because it is an excellent source of good fats and proteins (∼80% of seed content). Peanut has also become one of the most important contributors to human health and food security (Konate et al., 2020). In addition to cultivated peanuts, some wild species including *Arachis glabrata*, *Arachis pintoi*, *Arachis stenosperma*, and *Arachis villosulicarpa, etc*. are also used as food and medicine (Stalker, 2017). More importantly, some wild *Arachis* species possess many agronomic traits, such as disease and pest resistances (Subrahmanyam et al., 2001; Tallury et al., 2014), which are important in crop improvement, but these traits are not present in cultivated species (Upadhyaya et al., 2011). Although progress has been made through conventional breeding, yet the confusing species barrier between cultivated peanuts and wild species makes the utilization of genetic resources very difficult. The lack of a robust phylogeny of the *Arachis* genus has impeded the advances in basic biological research and molecular breeding of the cultivated peanuts.

Allotetraploidy, which are evident in soybean, *Brassica*, wheat, cotton, and peanut via whole chromosomal genome (Gill et al., 2009; Feldman et al., 2012; Paterson et al., 2012; Chalhoub et al., 2014; Bertioli et al., 2019; Zhuang et al., 2019), plays a critical role in the evolving history of most domesticated crop species. However, how allotetraploids species (e.g., cultivated peanut) have evolved from their diploid parents remains largely unknown (Bertioli et al., 2020; Zhuang et al., 2020). The lack of information is caused by two possible reasons: (1) morphological and molecular phylogenetic studies are not efficient in distinguishing taxonomic species for some horticulture features may have resulted from domestication. (2) Genetic diversity introduced by multiple parental inheritance makes it difficult to detect homology among sequences. According to a few previous studies, cultivated peanuts are allotetraploid (AABB genome type) and derived from two diploids wild species by a recent hybridization event (Bertioli et al., 2019; Zhuang et al., 2019). Many studies suggest that *A. duranensis* Krapov. & W.C.Greg. (AA) and *Arachis ipaensis* Krapov. & W.C.Greg. (BB) are the progenitor species, which provide valuable genetic resources to *A. hypogaea* (Kochert et al., 1996; Koppolu et al., 2010; Bertioli et al., 2011, 2016). However, some other studies support that cultivated peanuts may have been derived from more than two progenitor species, including *Arachis diogoi* Hoehne (AA), *Arachis correntina* (Burkart) Krapov. & W.C.Greg. (AA), *Arachis cardenasii* Krapov. & W.C.Greg. (AA), *A. batizocoi* Krapov. & W.C.Greg. (KK), *A. trinitensis* Krapov. & W.C.Greg. (FF), and *A. williamsii* Krapov. & W.C.Greg. (BB) (Stalker et al., 1991; Singh et al., 1994; Leal-Bertioli et al., 2014; Wang et al., 2019; Zhuang et al., 2019). The origination and evolution of the cultivated peanut species remains elusive, and it is extremely difficult to demarcate the boundary of some peanut species due to gene introgression, ancestral polymorphism and various speciation rates in different species (Moretzsohn et al., 2013; Bertioli et al., 2019).

The previous classification has put cultivated peanuts into two groups, subsp. *hypogaea* and subsp. *fastigiata*, based on some morphological and physiological characteristics, such as the presence of flower on main stem, time of maturation, the presence of seed dormancy, *etc.* (Gibbons et al., 1972; Krapovickas et al., 2007; Belamkar et al., 2011). According to some early classification work, which studied the growth habit, leaflet surface, branching pattern and pod traits of various peanuts (Ferguson et al., 2004; Krapovickas et al., 2007), subsp. *hypogaea* contain two botanical varieties, var. *hypogaea*, var. *hirsute*, while four varieties (var. *fastigiata*, var. *peruviana*, var. *vulgaris*, and var. *aequatoriana*) are present in subsp. *fastigiata*. However, classification based on morphological and physiological characteristics is not consistently supported by works done at the molecular level when employing different methods or using different genetic markers (He and Prakash, 2001; Gimenes et al., 2002; Moretzsohn et al., 2004; Koppolu et al., 2010). A molecular analysis using the AFLP approach shows that var. *aequatoriana* and var. *peruviana* are closely related to subsp. *hypogaea* (He and Prakash, 2001). Furthermore, a study carried out with SSRs markers put var. *peruviana* into subsp. *hypogaea*. More interestingly, var. *hypogaea* and var. *hirsute*, which are originally placed in subsp. *hypogaea*, are not even closely related according to Ferguson et al. (2004). The conventional classification of cultivated peanuts is supported by one recent study, which looked at high-quality SNPs in the peanut nuclear genomes (Zheng et al., 2018). However, the taxonomic boundaries among some botanical varieties cannot be clearly delimited in this study. Var. *hypogaea* and var. *hirsute* could not be distinguished due to difficulties in putting different accessions of the same variety into one cluster. A close evolutionary relationship was inferred between var. *hirsute* and var. *vulgaris* when using the plastomics approach (Wang et al., 2018, 2019). This study also supports a close relationship between var. *hypogaea* and var. *fastigiata*, which is different from what we would expect based on the previous classification. It seems that nuclear genomic sequence data is not sufficient or reliable in interpreting evolutionary relationship among allotetraploid species. Due to the lack of consistency, a study carried out with a different type of sequence data (i.e., plastomic data) or employing various analytic methods would be appropriate when trying to reconstruct the phylogeny of cultivated peanuts.

Plastomics provide a powerful tool in phylogenetic studies involving particular evolutionary events, such as interspecific hybridization, allopolyploidization, rapid evolution, *etc.* (Moore et al., 2007). In contrast to nuclear genomes, plastomes are maternally inherited. The evolutionary rate of plastomes is low, and there is no recombination during chloroplast division (Daniell et al., 2016). Therefore, plastomes are good resources for studying maternal evolutionary dynamics (Tonti-Filippini et al., 2017). Chloroplast genomes are highly conserved in angiosperms, which share a quadripartite structure containing a large single copy (LSC; 80–90 kb) and a small single copy (SSC; 16–27 kb) separated by two inverted repeats (IR; 20–28 kb) (Daniell et al., 2016). In green plants, plastomes typically range from 120 to 218 kb in size (Wicke et al., 2011), and such a variety in size is mainly caused by IR contraction and expansion (Choi et al., 2020; Henriquez et al., 2020). To take an extreme example, the IR region is completely lost in *Erodium* L’Herit. and some papilionoid legumes (Blazier et al., 2016; Lee et al., 2021). Angiosperm plastomes generally encode 110–130 genes, which include approximately 80 protein coding genes, 30 transfer RNA genes, and four ribosomal RNA genes (Daniell et al., 2016). Even though the loss of genes (Song et al., 2017; Alqahtani and Jansen, 2021) or introns (Jansen et al., 2007), and pseudogenization (Abdullah et al., 2021a; Li et al., 2021) have been reported in the plastomes of diverse plant species, plastomics still provide a reliable tool in phylogenetic studies, and plastid genomes have been largely used to reconstruct the phylogeny of many crop and horticulture species in recent years (Li et al., 2017; Xue et al., 2019; Guo et al., 2020; Hassoubah et al., 2020; Moner et al., 2020; Tyagi et al., 2020). However, there are only a limited number of peanut plastomes that have been sequenced and analyzed to date, including that of *A. hypogaea* and a few other related wild species (Prabhudas et al., 2016; Yin et al., 2017; Wang et al., 2018, 2019, 2021). This is insufficient in gaining a full picture of what has happened in the evolutionary history of cultivated peanuts and some wild species, and the relationship between cultivated peanuts and their potential wild maternal progenitor species is still unclear.

In this study, we assembled 33 *Arachis* plastomes including both cultivated and wild peanut species. Through comparative analysis with other peanut plastomes, which are currently available at NCBI, we aim to provide insights into species delimitation of *Arachis* and to identify the potential maternal genome progenitor species of cultivated peanuts. This work will serve as a foundation for the utilization of peanut genetic resources and the development of high-quality peanut varieties through molecular breeding.

## Materials and Methods

### Plant Sampling

In this study, Fresh young leave samples of 33 peanut accessions (24 species) representing 11 different genome types were collected from Henan Academy of Agricultural Sciences, Zhengzhou, China (HNAAS) and used for further analysis (Table 1). These include five botanical varieties of *A. hypogaea*, var. *hypogae* (Lainongzao), var. *hirsute* (Bajisitanhuapi), var. *fastigiate* (PI493938), var. *peruviana* (NcAc17090), var. *vulgaris* (Yiya). Samples were stored immediately in a −80°C freezer prior to DNA extraction. All the voucher specimens were deposited to the Herbarium of Zhengzhou University (Supplementary Table 1).

**TABLE 1:** Complete plastome features of the 33 *Arachis* accessions.

Species	Strains Information	Section	Plastome Size (bp)	IR (bp)	LSC (bp)	SSC (bp)	Number of genes (PCGs/tRNA/rRNA)	GC content (%; IR/LSC/SSC)
*A. batizocoi*	PI 298639	Arachis	156,340	25,781	85,846	18,932	109 (76/29/4)	36.4 (42.9/33.8/30.2)
*A. cardenasii*	PI 475996	Arachis	156,394	25,824	85,946	18,800	109 (76/29/4)	36.4 (42.9/33.8/30.2)
*A. cardenasii*	PI 476014	Arachis	156,410	25,825	85,958	18,802	109 (76/29/4)	36.4 (42.9/33.8/30.2)
*A. cruziana*	PI 476003	Arachis	156,364	25,785	85,851	18,943	109 (76/29/4)	36.4 (42.9/33.8/30.2)
*A. decora*	Grif 7721	Arachis	156,247	25,757	85,739	18,994	109 (76/29/4)	36.4 (42.9/33.8/30.2)
*A. diogoi*	PI 276235	Arachis	156,377	25,824	85,933	18,796	109 (76/29/4)	36.4 (42.9/33.8/30.2)
*A. duranensis*	PI 475844	Arachis	156,392	25,824	85,948	18,796	109 (76/29/4)	36.4 (42.9/33.8/30.2)
*A. duranensis*	PI 468200	Arachis	156,424	25,824	85,964	18,812	109 (76/29/4)	36.4 (42.9/33.8/30.2)
*A. duranensis*	PI 468323	Arachis	156,433	25,824	85,968	18,817	109 (76/29/4)	36.4 (42.9/33.8/30.2)
*A. duranensis*	PI 219823	Arachis	156,383	25,825	85,937	18,796	109 (76/29/4)	36.4 (42.9/33.8/30.2)
*A. glandulifera*	PI 468336	Arachis	156,363	25,774	85,870	18,945	109 (76/29/4)	36.4 (42.9/33.8/30.2)
*A. herzogii*	PI 476008	Arachis	156,420	25,825	85,962	18,808	109 (76/29/4)	36.4 (42.9/33.8/30.2)
*A. hoehnei*	Grif 7682	Arachis	156,379	25,824	85,942	18,789	109 (76/29/4)	36.4 (42.9/33.8/30.2)
*A. hypogaea* var. *fastigiata*	PI493938	Arachis	156,384	25,825	85,938	18,796	109 (76/29/4)	36.4 (42.9/33.8/30.2)
*A. hypogaea* var. *hirsuta*	Bajisitanhuapi	Arachis	156,387	25,825	85,942	18,795	109 (76/29/4)	36.4 (42.9/33.8/30.2)
*A. hypogaea* var. *hypogae*	Lainongzao	Arachis	156,387	25,825	85,942	18,795	109 (76/29/4)	36.4 (42.9/33.8/30.2)
*A. hypogaea* var. *peruviana*	NcAc17090	Arachis	156,387	25,825	85,942	18,795	109 (76/29/4)	36.4 (42.9/33.8/30.2)
*A. hypogaea* var. *vulgaris*	Yiya	Arachis	156,384	25,825	85,938	18,796	109 (76/29/4)	36.4 (42.9/33.8/30.2)
*A. ipaensis*	–	Arachis	156,394	25,776	85,904	18,938	109 (76/29/4)	36.4 (42.9/33.8/30.2)
*A. kempff-mercadoi*	PI 468330	Arachis	156,429	25,824	85,965	18,816	109 (76/29/4)	36.4 (42.9/33.8/30.2)
*A. microsperma*	PI 674407	Arachis	156,326	25,787	85,939	18,813	109 (76/29/4)	36.4 (42.9/33.8/30.2)
*A. monticola*	PI 263393	Arachis	156,388	25,824	85,945	18,795	109 (76/29/4)	36.4 (42.9/33.8/30.2)
*A. monticola*	PI 219824	Arachis	156,388	25,824	85,945	18,795	109 (76/29/4)	36.4 (42.9/33.8/30.2)
*A. palustris*	PI 666093	Arachis	156,220	25,827	85,750	18,907	109 (76/29/4)	36.4 (42.9/33.8/30.2)
*A. simpsonii*	Grif 14534	Arachis	156,385	25,824	85,941	18,796	109 (76/29/4)	36.4 (42.9/33.8/30.2)
*A. trinitensis*	PI 666101	Arachis	156,354	25,776	85,860	18,941	109 (76/29/4)	36.4 (42.9/33.8/30.2)
*A. valida*	PI 666103	Arachis	156,369	25,774	85,877	18,944	109 (76/29/4)	36.4 (42.9/33.8/30.2)
*A. villosa*	PI 298636	Arachis	156,464	25,824	85,958	18,858	109 (76/29/4)	36.4 (42.9/33.8/30.2)
*A. pintoi*	–	Caulorrhizae	156,311	25,757	85,736	18,970	109 (76/29/4)	36.4 (42.9/33.9/30.3)
*A. dardonoi*	–	Heteranthae	156,630	25,857	85,990	18,926	109 (76/29/4)	36.3 (42.9/33.8/30.2)
*A. pusilla*	PI 497572	Heteranthae	156,476	25,862	85,889	18,863	109 (76/29/4)	36.3 (42.9/33.8/30.2)
*A. rigonii*	PI 262142	Procumbentes	156,476	25,862	85,889	18,863	109 (76/29/4)	36.3 (42.9/33.8/30.2)
*A. glabrata*	PI 468366	Rhizomatosae	156,428	25,824	85,969	18,811	109 (76/29/4)	36.4 (42.9/33.8/30.2)

### Genomic DNA Extraction and Sequencing

Total genomic DNA of the 33 samples were extracted with the Tiangen Plant Genomic DNA Kit (Tiangen Inc., China) following the protocol provided by the manufacturer. DNA purity was assessed using the Qubit 2.0 (Invitrogen Inc., United States) and a NanoDrop machine (Thermo Scientific Inc., United States). DNA libraries were constructed using the Illumina Paired-End DNA library Kit and sequenced with a NovaSeq 6000 platform (Illumina Inc., United States) with a paired-end read length of 150 bp (NovoGene Inc., China). Upon completion, more than 6.0 GB raw reads were retrieved for each sample. The GetOrganelle toolkit was used for *de novo* assembling of the complete plastid genomes (Jin et al., 2020). The published plastomic sequences of *Arachis* (Supplementary Table 1) from GenBank were used as the seed file (“embplant_pt”) for the assembling process, as well as a template to estimate the possible circular sequence pattern.

### Plastome Annotation and Comparison

The Plastid Genome Annotator (PGA) software (Qu et al., 2019) was employed in the annotation of the selected peanut plastomes using *A. hypogaea* (accession no. MT712165) as a reference. The accuracy of annotation was evaluated with GeSeq (Tillich et al., 2017), HMMER (Wheeler and Eddy, 2013), and tRNAscan-SE (Lowe and Eddy, 1997) programs implemented in the CHLOROBOX web toolbox^1^ with a default setting. Chloroplot was used to visualize the plastid genomes as a physical map (Zheng et al., 2020). MISA-web (Beier et al., 2017) was used to identify simple sequence repeats (SSRs) with the following criteria: 10, 5, 4, 3, 3, and 3 repeat units are for mono-, di-, tri-, tetra-, penta-, and hexa-nucleotides, respectively. In addition, forward, palindrome, reverse, and complement repeated elements were identified using REPuter (Kurtz et al., 2001) with a minimal length of 30 bp, an identity value of more than 90% and a Hamming distance of 3. The comparison among whole chloroplast genomes in genus *Arachis* species were using data from 33 new sequenced plastomes, and published plastomes of five cultivated peanuts (Prabhudas et al., 2016; Wang et al., 2018), 12 wild peanuts (Wang et al., 2019) which downloaded from the NCBI database (Supplementary Table 1). Nucleotide diversity (Pi) of the plastomic sequences of *Arachis* species were obtained in this study and the published sequences were calculated using a sliding window method with a window length of 600 bp and a step size of 200 bp by DnaSP (Rozas et al., 2017).

### Phylogenetic Analysis

To reconstruct the phylogeny of peanut species and to identify the potential maternal progenitor species, the complete plastomes of 53 species (Supplementary Table 1) were retrieved from various databases and used to make a multiple sequence alignment with MAFFT under a default setting (Katoh and Standley, 2013). Among these species, *Dalbergia hupeana* Hance from the Tribe Dalbergieae was defined as outgroup. Phylogenetic trees were constructed with the 53 sequences using both the Maximum likelihood (ML) and the Bayesian inference method (BI), which are implemented in IQ-TREE (Nguyen et al., 2014) and MrBayes (Ronquist et al., 2012), respectively. The best fit nucleotide substitution models, TVM + F + R3 for ML analysis and GTR + F + I + G4 for BI analysis, were selected using the ModelFinder (Kalyaanamoorthy et al., 2017) according to the AIC criterion. In the ML analysis, 50,000 bootstrap replicates were carried out with the SH-aLRT branch test. The BI analysis was performed with two independent Markov Chain Monte Carlo chains with 2,000,000 generations, and it was considered to be stationary when the average standard deviation of split frequencies fell below 0.01. The first 25% of trees were discarded as burn-ins, and the remaining trees were used to construct a consensus tree.

## Results

### Characterization of the Peanut Plastomes

**FIGURE 1:**
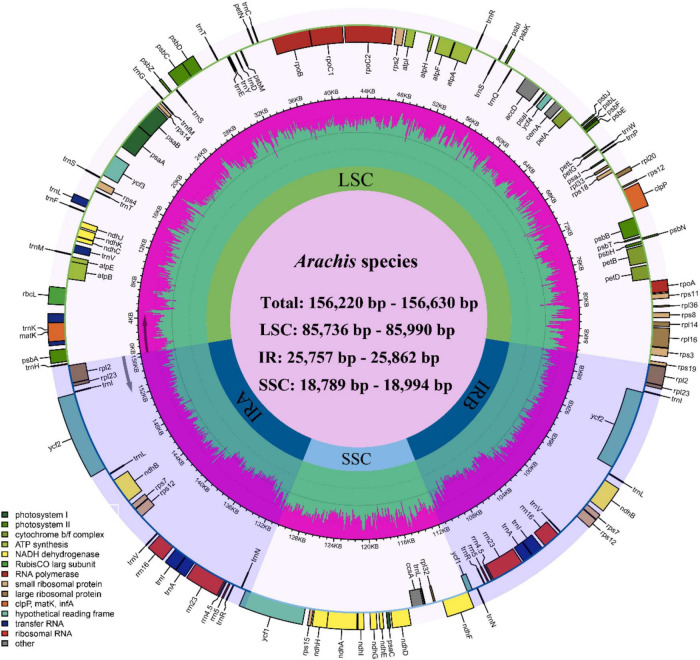
Circular plastome map of *Arachis*. Genes of different functional groups are color coded. The green in the inner circle corresponds to the GC content, while the pink corresponds to the AT content.

The size of the studied *Arachis* plastomes ranges from 156,220 bp (*Arachis palustris*) to 156,630 bp (*Arachis dardonoi*) in length (Table 1 and Figure 1), while the *Arachis* species ranging from 156,220 bp (*A. palustris*) to 156,878 bp (*A. hypogaea* var. *hirsute* AHL) in whole chloroplast genome length. Moreover, the cultivated peanut plastomes ranges from 156,354 to 156,878 bp in length, with *A. hypogaea* var. *hirsute* AHL being the largest. There is a 10–100 bp difference in length when comparing our sequencing data with the published data of a few species including *A. batizocoi*, *A. cardenasii*, *A. duranensis*, *A. ipaensis*, *Arachis villosa*, and *A. monticola* PI 219824. All the sequenced plastomes share a G + C content of 36.4% except for *A. dardonoi*, *A. pusilla*, and *A. rigonii*, which share a G + C content of 36.3%. All peanut plastomes contain a large single-copy (LSC), a small single-copy (SSC), and two inverted repeats (IRa/IRb). The LSC regions range from 85,736 bp (*A. pintoi*) to 85,990 bp (*A. dardonoi*) in length, with the G + C contents falling between 33.8 and 33.9%. The SSC regions vary from 18,789 bp (*Arachis hoehnei*) to 18,994 bp (*Arachis decora*) in length and the G + C content falls between 30.2 and 30.3%. *A. pintoi* has the smallest IRs, which is 25,757 bp in length, while a maximum IR length of 25,862 bp was observed in both *A. pusilla* and *A. rigonii.* These regions have a G + C content of 42.9%, which is significantly higher than that of the LSCs and SSCs. All plastomes included in our studies contain 109 unique genes, encoding 76 protein genes, 29 tRNAs, and 4 rRNAs (Table 1 and Figure 1), which is comparable with some well-studied *Arachis* species. Based on their annotated functions, these genes can be classified into four categories (Table 2), namely self-replication genes, photosynthesis related genes, other genes, and unknown function genes.

**TABLE 2:** List of the annotated genes in the plastomes of the selected *Arachis* accessions.

Category	Gene group	Gene name
Photosynthesis related genes	Rubisco	rbcL
	Photosystem I	psaA, psaB, psaC, psaI, psaJ
	Assembly/stability of photosystem I	ycf3
	Photosystem II	psbA, psbB, psbC, psbD, psbE, psbF, psbH, psbI, psbJ, psbK, psbL, psbM, psbN, psbT, psbZ
	ATP synthase	atpA, atpB, atpE, atpF, atpH, atpI
	Cytochrome b/f complex	petA, petB, petD, petG, petL, petN
	Cytochrome c synthesis	ccsA
	NADPH dehydrogenase	ndhA, ndhB(×2), ndhC, ndhD, ndhE, ndhF, ndhG, ndhH, ndhI, ndhJ, ndhK
Transcription and translation related genes	Transcription	rpoA, rpoB, rpoC1, rpoC2
	Ribosomal proteins	rpl14, rpl16, rpl2(×2), rpl20, rpl23(×2), rpl32, rpl33, rpl36, rps11, rps12(×2), rps14, rps15, rps18, rps19, rps2, rps3, rps3, rps4, rps7(×2), rps8
RNA genes	Ribosomal RNA	rrn4.5(×2), rrn5(×2), rrn16(×2), rrn23(×2)
	Transfer RNA	trnA-UGC(×2), trnC-GCA, trnD-GUC, trnE-UUC, trnF-GAA, trnfM-CAU, trnG-UCC, trnH-GUG, trnI-CAU(×2), trnI-GAU(×2), trnK-UUU, trnL-CAA(×2), trnL-UAA, trnL-UAG, trnM-CAU, trnN-GUU(×2), trnP-UGG, trnQ-UUG, trnR-ACG(×2), trnR-UCU, trnS-GCU, trnS-GGA, trnS-UGA, trnT-GGU, trnT-UGU, trnV-GAC(×2), trnV-UAC, trnW-CCA, trnY-GUA
Other genes	RNA processing	matK
	Carbon metabolism	cemA
	Fatty acid synthesis	accD
	Proteolysis	clpP
Genes of unknown function	Conserved reading frames	ycf1(×2), ycf2(×2), ycf4

*×2 denotes two gene copies in the IR region.*

### Comparative Plastomic Analysis

**FIGURE 2:**
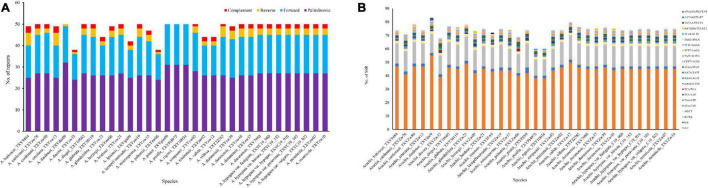
Analyses of repeated sequences in the plastomes of the 33 *Arachis* accessions. **(A)** Numbers of repeats and their types; **(B)** number of identified SSRs motifs and their types.

Analysis of the 33 new sequenced plastomes revealed 1,593 tandem repeats with complement, forward, reverse, and palindromic elements (>30 bp). The number of repeats present in each plastome varies considerably, ranging from 38 in *A. decora* to 50 in most other species (Figure 2A). In average, 17 forward, 27 palindromic, 2 complement, and 3 reverse repeats were estimated in each plastome. Among the species, in which repeats were identified, *A. dardonoi* lacks complement repeats, while *A. pintoi*, *A. pusilla*, and *A. rigonii* do not have complement or reverse repeats. Most repeats among *Arachis* species plastomes are present in the intergenic spacer regions. With the MISA analysis, 60 universal SSR loci were detected in the plastomes of *A. pusilla* and *A. rigonii* while 83 was in *A. dardonoi* (Figure 2B). Based on the SSR analysis, 40–57 of the identified SSRs are mononucleotidic, 14–20 are dinucleotidic, 1–4 are trinucleotidic, and 5–9 are tetranucleotidic (Supplementary Table 2). Among these SSRs, most of the identified mononucleotidic SSRs are composed of A/T, and the dinucleotidic ones contain AT/TA. Moreover, the pentanucleotidic SSRs in *A. dardonoi* and *A. ipaensis* have a typical sequence of AATAG/CTATT or TATAA/TTATA, and the hexanucleotidic SSRs in *A. cardenasii*, *A. dardonoi*, *A. duranensis*, *A. glabrata*, *Arachis herzogii*, and *Arachis microsperma* contain either AATGGA/TCCATT or ATAGCA/TGCTAT (Figure 2B).

**FIGURE 3:**
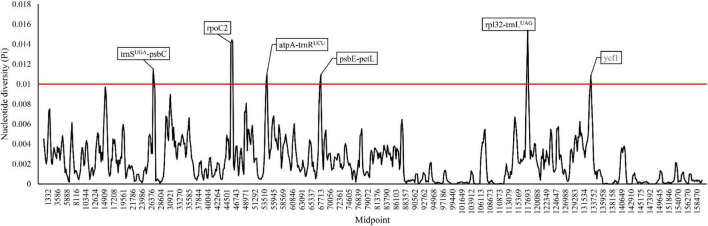
Sliding window analysis of the whole plastomes of the 52 *Arachis* accessions.

A total number of 3,416 polymorphic sites (Pi: 0.227%) were detected in the 52 cultivated and wild peanut plastomes (Supplementary Table 1), including 1,670 singleton variable sites and 1,746 parsimony informative sites. The alignment of seventeen peanut complex (see discussion) sharing high sequence similarity revels 54 singleton variable sites and 20 parsimony informative sites, which are also highly conserved across all the analyzed plastomes. Pi values among different plastomes were computed by a sliding window method with a window length of 600 bp and a step size of 200 bp (Figure 3). In addition, six hotspot regions with high Pi values were identified among various cultivated peanut accessions and other wild species, which include two protein-coding genes (*rpoC2* and *ycf1*) and four intergenic spacer regions (*trnS-UGA-psbC*, *atpA-trnR-UCU*, *psbE-petL*, and *rpl32-trnL-UAG*). These regions could be potentially used as DNA markers in phylogenetic studies of different *Arachis* species.

### Phylogeny of *Arachis* Based on Whole Plastomes

**FIGURE 4:**
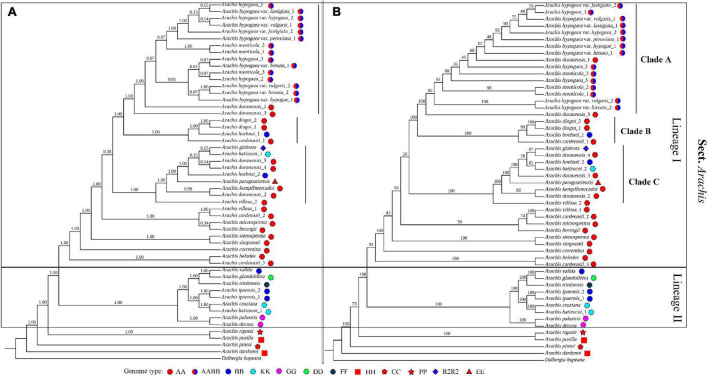
Phylogenetic trees constructed with 52 *Arachis* plastomic sequences using the Bayesian inferred **(A)** and Maximum likelihood **(B)** methods. Numbers above the branch represent the confidence level.

In our study, *Arachis* is recovered as monophyletic, which is well supported by both ML and BI analyses (Figures 4A,B). The basal position of *A. dardonoi* (HH) from section *Heteranthae* is strongly supported by both methods. Within section *Arachis*, two major lineages, Lineage I and II, were clearly defined (Figure 4). Another species *A. pusilla* (HH) from section *Heteranthae* is grouped into one clade with *A. rigonii* (PR) from section *Procumbentes*. However, ML and BI analysis did not provide consistent result in terms of the taxonomic statuses of *A. duranensis*, *A. monticola*, and cultivated peanut (Figures 4A,B).

**FIGURE 5:**
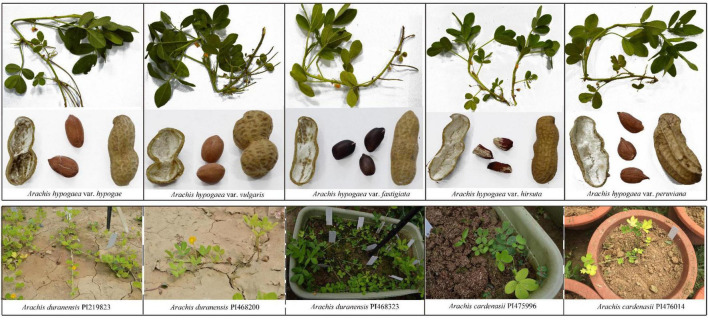
Morphological differences among selected *Arachis* species (three accessions of *A. duranensis*, two accessions of *A. cardenasii* and five cultivated peanuts).

Species with a genome type of AA are mainly distributed in Lineage I, which are further divided into three clades. Based on the BI analysis, the newly sequenced peanut varieties, var. *hypogaea* and var. *hirsuta*, are clustered into one clade, while var. *fastigiata*, var. *vulgaris*, and var. *peruviana* are placed in another clade with relatively low bootstrap values (Figure 4A). In the ML analysis, the six varieties are placed in one big clade, and it is impossible to draw a clear boundary between subsp. *hypogaea* and subsp. *fastigiated* (Figure 4B). Var. *vulgaris* (Yiya vs. AHZ) and var. *hirsute* (Bajisitanhuapi vs. AHL) are placed in two separate clades in this study. The cultivated peanuts and two wild species, *A. monticola* and *A. duranensis* (PI219823 and PI 475844), are grouped together as the “peanut complex” clade (Clade A), members of which demonstrate a diversity in morphological features, and the boundary between Clade A and other clades is not well defined or supported by the phylogenetic analyses (Figure 5). Both the ML and BI analyses support that *A. duranensis* (AA) is the wild diploid progenitor of all cultivated peanuts. In Lineage I, Clades B and C are not monophyletic, which contain species with various genome types, such as *A. hoehnei* (BB), *A. glabrata* (R2) from section *Rhizomatosae*, *A. batizocoi* (KK), and *Arachis paraguariensis* (EE) from section *Erectoides* (Figure 4).

The phylogenetic structure of Lineage II is strongly supported by both ML and BI analyses. It contains nine *Arachis* species/accessions with diverse genome types. For example, the two accessions of *A. ipaensis* and *Arachis valida* contain BB genome type. *A. decora* and *A. palustris* (2*n* = 18), share a genome type of GG and are placed together with high confidence scores. *A. valida* shows a sisterhood relationship with *Arachis trinitensis* (FF) and *Arachis glandulifera* (DD), while *A. ipaensis*, another possible diploid progenitor of cultivated peanuts, is grouped together with *A. batizocoi* (AA) and *Arachis cruziana* (KK).

## Discussion

### *Arachis* Plastomes Are Highly Conserved

All *Arachis* plastomes share a typical quadripartite structure, consisting of one LSC region and one SSC regions separated by a pair of IRs. The same structure has also been reported in other angiosperms (Xu et al., 2015; Daniell et al., 2016; Tonti-Filippini et al., 2017). All the *Arachis* plastomes covered in this study are highly conserved in genome size and structure, G + C content, and gene number, which are also comparable to the plastomes of previously published *Arachis* species (Prabhudas et al., 2016; Yin et al., 2017; Wang et al., 2018, 2019). Plastomes of angiosperms tend to vary in size, the size of a typical *Arachis* plastome is approximately 156 kb (Supplementary Table 1), similar with the plastomes length of soybean (*Glycine*) in 152 kb, but more than the length of wheat (*Tribe Triticeae*), which varies from 133 to 137 kb (Middleton et al., 2014), rice (*Oryza*) of 135 kb in size (Asaf et al., 2017), and less than buckwheat (*Fagopyrum*) of 159 kb in total length (Wang et al., 2017). Genome size change was suggested to be linked variation of intergenic region, InDel events and oligonucleotide/microsatellites repeats within the related species, while gene loss, expansion/contraction of an IR region among seed plants (Xu et al., 2015; Zheng et al., 2017).

All the published *Arachis* plastomes share the same number of protein coding genes (Table 2) with only a few exceptions. Prabhudas et al. (2016) was not able to detect NADH dehydrogenase subunit 2 gene (*ndhB*) in *A. hypogaea* Co7, and *orf42* and *ycf68* were miss annotated in another two studies by Yin et al. (2017) and Wang et al. (2019). A closer look at the coding regions reveals that five tRNAs and 11 protein coding genes harbor at least one intron. Among these, *ycf3*, *clpP*, and *rps12* (a *trans*-splicing gene) contain two introns (Xu et al., 2015; Liu et al., 2020). The total number of tRNA genes present in our sequenced plastomes is 29, and the same conclusion was reached in two other studies by Schwarz et al. (2015) and Wang et al. (2019). However, one extra tRNA gene was annotated in one previous study carried out by Prabhudas et al. (2016). This one extra gene is *trnP*-GGG, which overlaps with another tRNA gene^2^. According to wild Roses, *trnP*-GGG gene in the region of *trnP*-UGG gene also exists (Jeon and Kim, 2019). Former studies demonstrated a widely distributed of *trnP*-GGG gene present in charophyte to gymnosperm, while *trnP*-UGG gene in plastomes from algae to higher plants (Turmel et al., 2002; Sugiura and Sugita, 2004).

Microsatellites and oligonucleotide repeats play an important role in the identification of regions with a large number of mutations, and are helpful in the study of population genetics (Ahmed et al., 2012; Abdullah et al., 2019). A consistent result was obtained when comparing SSRs and oligonucleotide repeats across different *Arachis* plastomes (Yin et al., 2017; Wang et al., 2019), with A/T and AT/TA being the most common mononucleotidic SSRs and mononucleotidic SSRs, respectively. A similar pattern is also reported in plastomes of many other angiosperms (Tian et al., 2019; Mehmood et al., 2020; Abdullah et al., 2021b). The SSRs loci identified in this work could serve as potential molecular markers for understanding the population genetic structure among various *Arachis* species. Here, we also identified some oligonucleotide repeats, which are associated with nucleotide substitution, mutation and InDel events in the genomes (Abdullah et al., 2021b,c). Most of the oligonucleotide repeats were found in the intergenic regions, and a similar pattern is observed in the plastomes of many other vascular plants (Kuang et al., 2011; Li et al., 2017; Sigmon et al., 2017; Wang et al., 2019). Our results also showed a high abundance of complement and forward oligonucleotide repeats across different *Arachis* species. Oligonucleotide repeats could be used for the identification of regions with mutations and the reconstruction of accurate phylogeny of *Arachis* species (Mehmood et al., 2020; Abdullah et al., 2021b).

### Linking Phylogeny With Genome Type

The genus *Arachis* consists of 81 species demonstrating a huge diversity in genome types (A, B, AB, C, D, E, EX, F, H, K, PR, R1, R2, T, and TE). Linking phylogenetic analysis with their genome type information could allow us to better understand the origination and evolution of cultivated peanuts. Based on our study, hybridization seems to play a major role in the evolution history of cultivated species (Garcia et al., 1995; Jarvis et al., 2003). However, problems within several clades are still unsolved. Our results show that the taxonomic relationship based on morphology should be revised (He et al., 2014; Vishwakarma et al., 2017). Two studies working with plastomics data (Wang et al., 2019) and microsatellite markers (Moretzsohn et al., 2013) also reached the same conclusion. In addition, one clade may contain species with various genome types, which is supported by this study and two other phylogenetic studies working with intron sequences and microsatellite markers (Moretzsohn et al., 2013). Again, it is very difficult to delimit the boundary of different *Arachis* species. In fact, all *Arachis* species look very similar morphologically, and leaf shape could probably be the only morphological trait, which could potentially be used in putting species into different taxonomic groups (Supplementary Figure 1). We speculate that recent speciation events play an important role in the evolution of *Arachis*. Both underground fruiting and clistogamy are thought to limit gene flows and seed dispersal in peanuts (Tan et al., 2010; Zhang et al., 2017), which should allow each species to keep its distinct identity (Yu et al., 2020). However, it is very interesting to see that the flowers and stems of *Arachis* plant could attract small insects, such as ants (Supplementary Figure 2). The movement of ants between different plants could cause the pollen of one species to be transferred to another species, and therefore promote gene flow between different *Arachis* species. In fact, genome introgression was detected among the interspecific hybrid population of peanuts (Garcia et al., 1995).

Plastomes are highly conserved and tend to have low nucleotide variations (Sigmon et al., 2017; Wang et al., 2018; Nock et al., 2019) (Figure 4). In this study, only 74 nucleotide polymorphisms were detected among different species of the cultivated peanut complex, indicating that the plastomes of cultivated peanuts are highly conserved (Wang et al., 2018). This observation could also be explained with a low nucleotide substitution rate. Peanut has only been domesticated for several thousand years, there is not enough time to accumulate many genetic variations (Bertioli et al., 2019). Although most botanical varieties examined in this study do demonstrate differences in their morphology (Figure 5), there are no distinguishable morphological features, which could be used to put different species into the two subspecies groups. For example, var. *fastigiate*, var. *vulgaris* and var. *hirsute* coming from two different groups all have three or more seeds in each shell (Figure 4A). The overall phylogeny obtained in this study is in agreement with the conventional classification based on studies looking at other features, including morphology (Krapovickas et al., 2007), AFLP markers (He and Prakash, 2001), simple sequence repeats (Ferguson et al., 2004), and single nucleotide polymorphisms (Zheng et al., 2018). However, violations do exist when it comes to the phylogenetic relationship of different varieties, such as, var. *peruviana* does not belong to subsp. *fastigiata* (He and Prakash, 2001; Ferguson et al., 2004). Var. *hypogaea* and var. *hirsute* should not be placed in subsp. *hypogaea* according to the conventional classification.

### Maternal Hybridization Event in the History of Cultivated Peanuts

Our results strongly support the hypothesis that *A. duranensis* is the wild diploid progenitor (with a genome type of A) of cultivated peanuts (Figure 4). This result is compatible with the earlier view, which is based on multiple lines of evidence from comparative genomics, geographic distribution, phylogenetic reconstruction, *etc.* (Kochert et al., 1996; Seijo et al., 2004; Fávero et al., 2006; da Cunha et al., 2008; Bertioli et al., 2016; Chen et al., 2016; Wang et al., 2019). Furthermore, phylogenomic investigation using both ML and BI methods suggests that *A. duranensis* have diverged into two groups. *A. duranensis* (PI219823 and PI 475844) shows a closer relationship with *A. hypogaea*, while *A. duranensis* PI 468200, PI 468323, and PI263133 (Genbank no. MK144822) are grouped in another clade containing *A. batizocoi*, *A. glabrata*, *A. hoehnei*, *Arachis kempff-mercadoi*, and *A. paraguariensis* (Figure 4). This topology was generally consistent with that of the ML tree, in which the three botanical accessions of *A. duranensis* (ICG 8138, ICG 8123, and PI 262133) are distributed in different clades (Zhuang et al., 2019). Moreover, the 42 accessions of *A. duranensis* demonstrate clear variations in morphological features (Singh et al., 1996). In agreement with Bertioli’s work (Bertioli et al., 2019), some accession of *A. duranensis* may have served as the AA sub-genome maternal progenitor of *A. hypogaea*. However, the status of *A. diogoi* (former known as *Arachis chacoensis*) and *A. cardenasii* as another two potential progenitors is not supported by our study.

This does not contrary to the earlier view that *A. monticola* is the direct progenitor of cultivated peanuts, and that it plays a vital role in the transition of diploid wild species to tetraploid cultivated species (Simpson et al., 2001; Yin et al., 2020). Cultivated peanut (*A. hypogaea*) and wild *A. monticola* are allotetraploids (AABB), while other 30 described wild species are diploid (Stalker, 2017). The previous phylogeographical analyses often group these two species (*A. hypogaea* and *A. monticola*) together (Gimenes et al., 2002; Seijo et al., 2004). As former documented, *A. monticola* is a weedy subspecies of cultivated peanuts, and it is placed in one group with *A. hypogaea* in earlier phylogenetic studies (Koppolu et al., 2010; Stalker, 2017; Vishwakarma et al., 2017; Wang et al., 2019). Their close relationship can be further supported with the following evidence. Firstly, *A. hypogaea* is able to produce fertile hybrids when hybridized with *A. monticola* (Stalker and Moss, 1987). Secondly, this is in agreement with the results of previous studies focusing on somatic chromosomes, such as the virtually identical centromeric bands and *in situ* hybridization between *A. hypogaea* and *A. monticola* (Raina and Mukai, 1999). Thirdly, *A. monticola* may have been derived from a more ancient hybridization event according to the phylogenetic studies on the two *FAD2A* alleles, while the accessions of *A. hypogaea* may have evolved latter (Jung et al., 2003). During its evolution, *A. monticola* has accumulated more mutations in its plastome than most other cultivated peanuts do, which could be possibly traced back to different evolution rates or natural selection. Nevertheless, plastomics approach is very useful in inferring the maternal origin of cultivated peanuts and explaining the close phylogenetic relationship between *A. monticola* and *A. hypogaea*.

## Conclusion

In summary, 33 *Arachis* plastomes were sequenced and analyzed in a comparative framework with the published plastomics data of cultivated and wild peanut species. These plastomes share similar structural organization with low nucleotide variations. The phylogenetic topology obtained in this study shows that plastomics could facilitate a better understanding of the phylogeny among deep lineages of *Arachis*. Based on our result, it is speculated that cultivated peanuts have experienced a multi-maternal hybridization event with a recent origin. Some wild species of the *A. duranensis* accessions might have contributed the maternal sub genomes to cultivated peanuts and *A. monticol*a, which represents a transitional species between wild diploid species and tetraploid cultivated species. Owing to interspecific gene flow and recent speciation, the relationship among different *Arachis* species inferred based on phylogeny do not always go along with their genome types. As a result, more *Arachis* species with various genome types should be included in future study to fully elucidate the origin and evolutionary history of *Arachis*.

## Data Availability Statement

The datasets presented in this study can be found in online repositories. The names of the repository/repositories and accession number(s) can be found in the article/Supplementary Material.

## Author Contributions

ZW, XZ, and BH conceived the ideas. PD and LF contributed to the sampling. XT and LS performed the experiments. XT and YW analyzed the data. The manuscript was written and improved by XT, LS, JG, ZW, XZ, and BH. All authors contributed to the article and approved the submitted version.

## Conflict of Interest

The authors declare that the research was conducted in the absence of any commercial or financial relationships that could be construed as a potential conflict of interest.

## Publisher’s Note

All claims expressed in this article are solely those of the authors and do not necessarily represent those of their affiliated organizations, or those of the publisher, the editors and the reviewers. Any product that may be evaluated in this article, or claim that may be made by its manufacturer, is not guaranteed or endorsed by the publisher.

## Footnotes

1
https://chlorobox.mpimp-golm.mpg.de/geseq.html

2
http://www.ncbi.nlm.nih.gov/nuccore/KX257487
