# Supplementary material for: Chloroplast Phylogenomic Analyses Reveal a Maternal Hybridization Event Leading to the Formation of Cultivated Peanuts
Source: Front Plant Sci. 2021 Dec 17;12:804568. doi: 10.3389/fpls.2021.804568 (PMC8718879; doi:10.3389/fpls.2021.804568)

Supplementary Figures 1 The morphogenic characters of *Arachis* species

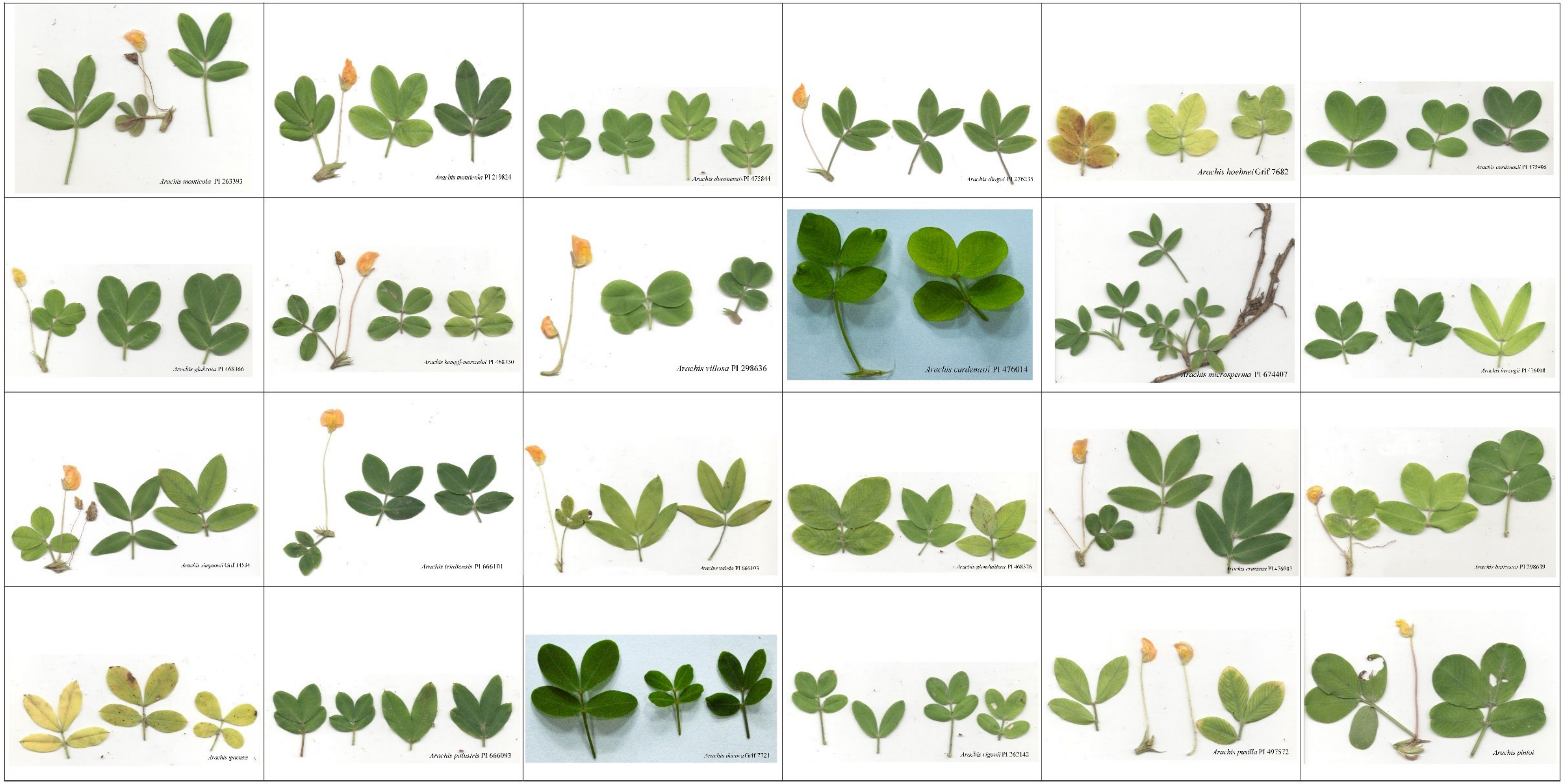

Supplementary Figures 2 Observation of ants on the flowers and stems of *Arachis* plants in the germplasm garden.

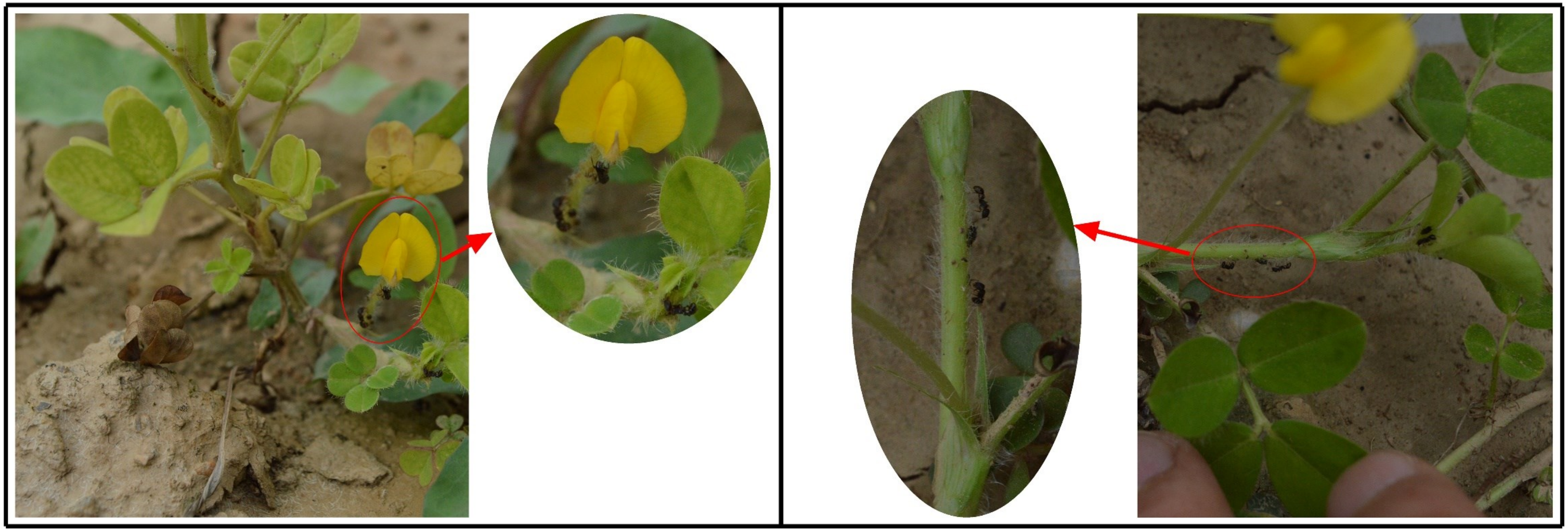

Supplement: Supplementary file 2 [file Image_1.pdf]
